# A qualitative assessment of the adverse effects associated with COVID-19 vaccines: a study from Jordan

**DOI:** 10.1186/s40545-023-00605-5

**Published:** 2023-08-10

**Authors:** Rawan Al-Bawab, Rana Abu-Farha, Faris El-Dahiyat, Razan I. Nassar, Mohammed Zawiah

**Affiliations:** 1 Department of Clinical Pharmacy and Therapeutics, Faculty of Pharmacy, Applied Science Private University, Amman, Jordan; 2 Clinical Pharmacy Program, College of Pharmacy, Al Ain University, Al Ain, United Arab Emirates; 3 AAU Health and Biomedical Research Center, Al Ain University, Al Ain, United Arab Emirates; 4 Department of Pharmacy Practice, Faculty of Clinical Pharmacy, Hodeidah University, Al Hodeidah, Yemen

**Keywords:** Side effects, COVID-19, Vaccine, Immunization, Jordan

## Abstract

**Objectives:**

The current study aimed to qualitatively explore the side effects reported by participants who received the COVID-19 vaccine among the Jordanian population.

**Methods:**

Between April 18th and May 12th, 2022, an in-depth interview was conducted with a purposive sample of vaccinated individuals to assess the side effects of the COVID-19 vaccine in this study. Thematic analysis was used to identify themes and sub-themes within the current qualitative data.

**Results:**

A total of 20 participants were interviewed. They had a mean age of 41.3 (SD = 14.3) years. Half of the participants were females (*n* = 10, 50.0%). The study revealed six main themes: first, most of the respondents believed that COVID-19 vaccines were safe. Second, the vaccines are not equivalent in their safety. The third there showed that participants follow preventive measures to decrease the possibility of experiencing side effects. The fourth theme showed that reporting of side effects by the participants is dependent on the experienced side effects. Moreover, the next theme revealed that participants showed hesitancy to take more than one type of vaccine. Finally, participants were willing to take the vaccine annually, because they believed that the vaccine is better than the disease itself and decreases the aggressive effects of the disease.

**Conclusions:**

This study found that the majority of participants believed in the safety of the COVID-19 vaccines and emphasized the responsibility of the healthcare providers in increasing awareness among the population about the importance of the vaccines. Enhancing such awareness is essential to improve the acceptance of receiving different types of vaccines.

## Background

The World Health Organization (WHO) declared the novel coronavirus a worldwide pandemic in March 2020 [1]. Since the coronavirus (COVID-19) is a highly contagious virus, the number of confirmed cases and death rates have increased dramatically [1, 2]. Several preventative measures were taken by the government to reduce the spread of the virus, including mandatory mask-wearing, social distancing, and national curfews [2]. Furthermore, governments worldwide have pinned their hopes on the development of COVID-19 vaccines [3–5].

Even though several medications have been reported to resist COVID-19, they remain to be supportive, and their potency and efficacy must still be determined by additional randomized control trials [3, 6, 7]. To date, no antiviral medication has shown a significant decrease in mortality. Therefore, the development of COVID-19 vaccines was essential, as it was the most efficient strategy to fight the pandemic. Global efforts were made to expedite the development and creation of COVID-19 vaccines [8].

As of December 2020, there were more than 200 vaccine candidates for COVID-19. Despite this, the public’s mistrust of COVID-19 vaccines has emerged as a significant barrier to vaccination [9]. Likewise, several individuals were hesitant to receive the COVID-19 vaccine due to concerns about its safety and efficacy, including the duration of COVID-19 protection, as several cases of reinfection have been documented [10]. The rapid development of COVID-19 vaccines is the main reason for such concerns about vaccine’s safety. Moreover, the occurrence of adverse events has caused some people to be more hesitant, delay or oppose vaccination [11]. In general, vaccine hesitancy is a result of a complicated decision-making process, influenced by factors, such as historical events, political factors, prior vaccination experience, and risk perception [12, 13].

The Food and Drug Administration has approved the use of several COVID-19 vaccines in an emergency situation. Among the licensed COVID-19 vaccines were Pfizer-BNT162b2, BioNTech’s AstraZeneca’s AZD-1222, Sinopharm’s BBIBP-CorV, Johnson & Johnson's Ad26.COV2.S, and Sputnik vaccines [14]. Due to the rapid development of these vaccines, it is crucial to investigate potential side effects that may arise after receiving them. By reporting and identifying post-vaccination symptoms, this study sheds light on the safety of COVID-19 vaccines, providing valuable information to clinicians and healthcare workers on potential long-term consequences. This way, these side effects can be appropriately prevented or managed. The current study aimed to qualitatively explore the side effects experienced by participants reported after receiving the COVID-19 vaccine among the Jordanian population.

Using the qualitative research method allows researchers to extensively explore individuals’ experiences, perceptions, and perspectives, and it has wide possibilities within the area of healthcare research [15]. Thus, the qualitative method was selected to understand participants’ experience and perspective of the COVID-19 vaccine's side effects; similar to other published studies [16, 17], and to assist in the identification of unknown or unreported COVID-19 vaccine side effects. By acquiring these details, the qualitative method contributes to a more comprehensive understanding of COVID-19 vaccine safety.

## Methods

### Study design, participants, and data collection

Between April 18th and May 12th, 2022, an in-depth interview was conducted with a non-probability convenience sample of vaccinated individuals to assess the side effects of the COVID-19 vaccine in this study. Appointments were scheduled by email and consent forms were sent to the participants, who were then interviewed using the Zoom application. Before recording each interview, permission was obtained from the participant. Interview guide questions were formed and developed by the investigators in English to ensure proper responses from the participants. The interviews were conducted using the participants’ mother tongue language (Arabic), and they were subsequently translated into English. All interviews were conducted by a single pre-trained researcher to reduce bias.

### Interview instruments

The guided questions included three major sections. The first section gathered general information about participants, including age, gender, educational level, and marital status. The second section focused on the history of COVID-19 infection, the type of COVID-19 vaccine received, and the number of received doses. The third section explored participants’ perception of COVID-19 vaccine safety, experiences with adverse effects, prevention and management of side effects, factors influencing post-vaccination side effects, and the willingness to take vaccines in the future.

### Ethical consideration

The approval on ethical consideration was obtained from the Institutional Review Board at Applied Science Private University (Approval number: 2022-PHA-9). The study followed the ethical standards outlined in the World Medical Association Declaration of Helsinki guideline [18]. Participants were informed that their participation in the study is voluntary and that their responses will be kept classified and evaluated only.

### Data analysis

Thematic analysis was the method used to identify themes or patterns within the current qualitative data [19], because it is flexible and considered the umbrella of many other types of analysis [20]. During thematic analysis, participants’ opinions, thoughts, and interactions were summarized into notes, which were then converted into themes based on similarities and relationships. Thematic analysis was independently performed by two researchers who reviewed the notes and identified all potential themes. The resultants’ themes were evaluated, and where necessary, renamed or grouped together due to their similarity.

### Trustworthiness

Criteria for trustworthiness was employed as outlined by Lincoln and Guba [21]. The credibility criterion was achieved through prolonged engagement with participants and data, the use of peer checking, and an enhanced thorough description of source data and detailed methods. Dependability was achieved through “peer checking”, where experienced authors re-analysed some of the data to ensure accurate analysis. Transferability was fulfilled by providing a detailed description of the study such as the process and participants to enable the reader to decide how the results may transfer.

### Data presentation

The participants were assigned a number based on the sequence in which they were interviewed. The findings were presented in the results section with supporting quotations from participants, which are indicated by their assigned number. Where appropriate, the percentage of participants and respective denominators (total or subgroup) for specific themes was presented.

## Results

### Sociodemographic characteristics of the study participants

**Fig. 1:**
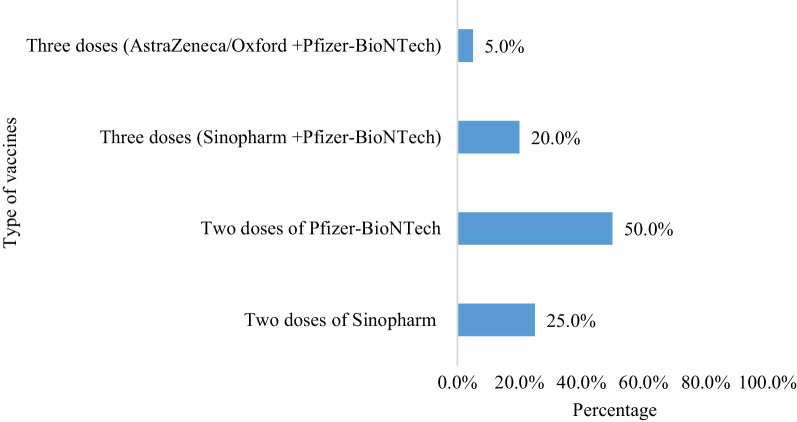
Distribution of COVID-19 vaccine types among the study participants (*n* = 20)

Twenty participants were interviewed and the duration of their interviews ranged from 5 to 15 min. Participants had a mean age of 41.3 (SD = 14.3) years, with half of them being female (*n* = 10, 50.0%). In addition, 75.0% of the participants (*n* = 15) were married and did not have medical-related degrees. Among them, 35.0% (*n* = 7) had not been infected with COVID-19 before receiving the vaccine. Three-quarters of the participants (*n* = 15, 75.0%) received two doses of the COVID-19 vaccine, while the remaining received three doses (25.0%). The demographic characteristics and COVID-19-related information of the participants are outlined in Table 1. In regards to the most commonly received COVID-19 vaccine among the participants (Fig. 1), it was observed that three-quarters of them received at least one dose of the Pfizer-BioNTech vaccine (*n* = 15, 75.0%), while nine participants (45.0%) received at least one dose of Sinopharm vaccine. Only one participant (5.0%) received the AstraZeneca/Oxford vaccine.

**Table 1:** Sociodemographic and health characteristics of the study sample (*n* = 20)

	Gender	Age	Educational level	Marital status	Number of vaccine doses received	Name of vaccine(s) received	Get infected with COVID-19 before receiving the vaccine	Medical-related degree
P1	Female	60	School level	Married	Two doses	Sinopharm	Yes	No
P2	Male	62	School level	Married	Two doses	Sinopharm	Yes	No
P3	Female	30	Bachelor	Married	Two doses	Pfizer-BioNTech	Yes	No
P4	Male	37	Bachelor	Married	Two doses	Sinopharm	No	No
P5	Female	32	Bachelor	Married	Two doses	Sinopharm	Yes	No
P6	Male	34	School level	Married	Three doses	AstraZeneca/Oxford (2) Pfizer-BioNTech (1)	Yes	No
P7	Male	69	School level	Married	Three doses	Sinopharm (2) Pfizer-BioNTech (1)	No	No
P8	Female	64	Diploma	Married	Three doses	Sinopharm (2) Pfizer-BioNTech (1)	No	No
P9	Male	32	School level	Single	Three doses	Sinopharm (2) Pfizer-BioNTech (1)	No	Yes
P10	Female	36	School level	Other	Two doses	Sinopharm	Yes	No
P11	Male	52	Diploma	Married	Three doses	Sinopharm (2) Pfizer-BioNTech (1)	Yes	No
P12	Male	27	Bachelor	Married	Two doses	Pfizer-BioNTech	No	Yes
P13	Female	44	Master	Married	Two doses	Pfizer-BioNTech	Yes	Yes
P14	Female	29	Master	Married	Two doses	Pfizer-BioNTech	No	Yes
P15	Male	34	Bachelor	Single	Two doses	Pfizer-BioNTech	Yes	No
P16	Male	45	School level	Married	Two doses	Pfizer-BioNTech	Yes	No
P17	Female	42	Diploma	Married	Two doses	Pfizer-BioNTech	Yes	No
P18	Female	52	School level	Married	Two doses	Pfizer-BioNTech	Yes	No
P19	Male	23	Bachelor	Single	Two doses	Pfizer-BioNTech	Yes	No
P20	Female	21	Diploma	Single	Two doses	Pfizer-BioNTech	No	Yes

### Thematic analysis

Qualitative data analysis was conducted to highlight main five domains: COVID-19 vaccines safety, differences between COVID-19 vaccines, prevention and management of COVID-19 vaccine side effects, willingness to report COVID-19 vaccine adverse effects, and future willingness to get vaccinated regularly. The main emerged themes and related sub-themes are listed in Table 2.

**Table 2:** List of emerging themes and sub-themes

Domain of questions	Main theme	Sub-themes
COVID-19 vaccines safety	COVID-19 vaccines are generally safe to be taken	COVID-19 vaccines were recommended by news, social media, and doctors
		Vaccines are associated with well-tolerated side effects
Differences between COVID-19 vaccines	COVID-19 vaccines are not equivalent in their safety	Safety is based on the manufacturer company
		Safety is based on the mechanism of action
Prevention and management of COVID-19 vaccines’ side effects	Follow preventive measure to decrease the possibility of experiencing side effects	Test for COVID-19 before taking the vaccines
		Avoid taking the COVID-19 vaccine while having any other infection
		Follow safety measures after taking the COVID-19 vaccine
		Consume vitamins, healthy food, and perform physical activity to increase the immunity
Willingness to report COVID-19 vaccines’ adverse effects	Reporting is dependent on the experienced side effects	Willingness to report severe and aggressive side effects
		Willingness to report unknown side effects
		Willingness to report long lasting side effects
Willingness to take the vaccine regularly in the future	Hesitancy to take more than one type of vaccine	Preferring a previously used vaccine
		The lack of awareness about using different types of vaccine
	Willingness to receive the vaccine annually	

#### First domine: COVID-19 vaccines safety

The only emerging theme regarding COVID-19 vaccine safety was that **COVID-19 vaccines are generally safe to be taken**, with two sub-themes. The first sub-theme was that ***COVID-19 vaccines were recommended by news, social media, and doctors.*** It was found that 60.0% (n = 12) of the participants agreed with this statement. For instance, participant 1 stated *“Yes, it is safe. I was convinced to take the vaccine after hearing about it on TV and doctors recommending it, furthermore, I had no fear or hesitation about receiving it.”*. Similarly, participant 3 said, *“Yes, I’ve decided to take the vaccine after I have heard from many healthcare providers, and the media (television) that COVID-19 vaccines are safe regardless of the type of vaccine, and if I become infected after receiving the vaccine”.*

The second sub-theme was that ***Vaccines are associated with weltolerated side effects.*** Most of the participants believed that the side effects that occurred following vaccination are less severe than the negative effects of COVID-19. *“Of course, I believe that no matter how severe the vaccine’s side effects are, it will be much better than getting infected. Most side effects from vaccinations (approximately 90%) were controlled and the patients rarely required hospital admission”* **P9.**

#### Second domine: differences between COVID-19 vaccines

The second emerged theme following qualitative analysis for the differences between COVID-19 vaccines was that **COVID-19 vaccines are not equivalent in their safety**, where 70.0% (n = 14) of the participants believed that there are differences in the efficacy and adverse reactions between the different types of COVID-19 vaccines. This theme includes two sub-themes. The first sub-theme is that ***Safety is based on the manufacturer company***. The participants believed that since each vaccine was manufactured in a different country by a different manufacturer, each one has its own unique production process, resulting in variances in the side effects of the vaccines. Some participants reported that the British and American vaccines are better than Chinese’ one. *“No, for me, COVID-19 vaccines were not the same, and it is possible that there may be differences in the side effects between the various types available in the market, depending on how the vaccine is manufactured, its efficiency, and how it interacts with each individual body” said* **P14.** *“Of course, COVID-19 vaccines are different in their side effects, strength, and efficacy. For example, Pfizer is a well-known company. When compared to other companies, I consider it the best, so I took their vaccine to be reassured” said* **P13.** *“No, vaccines certainly are not the same. The British AstraZeneca vaccine is an example of this. It causes clots, according to the media in Europe and around the world” said* **P4.**

The second sub-theme was that ***Safety is based on the mechanism of action***. The participants stated that COVID-19 vaccines have different mechanisms of action to enhance the immune system, and thus, the side effects are based on each mechanism. *“Each COVID-19 vaccine has a specific mechanism of action, and each type stimulates the immune system through a different unique mechanism”* **P9.** *“Certainly, vaccinations will differ, because each COVID-19 vaccine was developed by a different company and was produced using a different approach based on the mechanism by which the vaccines work”* **P12.**

#### Third domine: prevention and management of COVID-19 vaccines’ side effects

Regarding the prevention and management of COVID-19 vaccines’ side effects, the main theme is that **Follow preventive measure to decrease the possibility of experiencing side effects.** Most participants took different precautions before receiving the COVID-19 vaccines to prevent or at least decrease the side effects associated with the vaccines.

The first sub-theme was ***Test for COVID-19 before taking the vaccines***. Some participants tested for COVID-19 before taking the vaccine to avoid any aggressive side effects. *“Before taking the COVID-19 vaccine, I tested for COVID-19 and found that I am in good health condition, and do not have any infections. Immediately after that I went and took the vaccine”* **P1.** *“Like anyone, I tested for COVID-19 to be sure that I was not infected and did not have any infections”* **P2.**

The second sub-theme was ***Avoid taking the COVID-19 vaccine while having any other infection.*** *“When I went to get the vaccine, I was conscious of my good health, and certain there were no other diseases, such as the flu” P10.*

The third-sub theme was ***Following safety measures after taking the COVID-19 vaccine.*** *“After taking the vaccine, I avoid direct contact with others. Moreover, I wore a face mask to protect myself from getting corona like most people”* **P6.** *“I am convinced that avoiding social contact can help to lessen the vaccine's side effects” P13.*

The fourth-sub theme was ***Consuming vitamins, healthy food, and perform physical activity to increase the immunity***. Some participants believed that these factors would decrease the severity and occurrence of side effects. *“In addition to safety precautions, general hygiene, and limiting social interaction, persons who perform physical activity, consume health food, and take vitamins will have less the side effects”* **P15.** *“In all areas of life, a person who takes preventive measures, cares about his health, and plays sports will certainly be this person who likes to preserve his body and maintain his health”* **P16.** *“To ensure that the side effects of COVID-19 vaccine are mild, one should take vitamins, and eat a health food”* **P2.** *“Of course, eating a variety of healthy foods and drinking different beverages will strengthen the body's immune system, as well using herbs, hot tea, lemon, citrus, orange juice, and hot pepper”* **P11.**

#### Fourth domine: willingness to report adverse effects from COVID-19 vaccines

The only theme for willingness to report COVID-19 vaccines’ adverse effects was: **reporting is dependent on the experienced side effects,** which includes three sub-themes. 1) ***Willingness to report severe and aggressive side effects “I will report if the side effects are severe or may threaten my life”*** **P3.** *“If the side effects are very severe, such as fever (for example, above 38 °C) or general body fatigue and exhaustion, I will notify the Ministry of Health” P15. 2) Willingness to report unknown side effects “If I had encountered unfamiliar or uncommon symptoms, I should report the side effect” P12.* And **3) Willingness to report long-lasting side effects. “If the side effects last more than a day, they can be reported”** **P20** *“If the side effects continued for more than two days, I may alert my brother because he is a doctor or call the Ministry of Health's emergency line”* **P4.**

#### Fifth domine: willingness to take the vaccine regularly in the future

Regarding participants’ willingness to take the vaccines regularly in the future, two main themes were identified. The first theme was **Hesitancy to take more than one type of vaccine** and two sub-themes were included in this theme. The first sub-theme was ***Preferring a previously used vaccine***. It was found during the interviews that 65.0% of the participants (n = 13) were hesitant to take more than one type of the COVID-19 vaccines. *“I would rather take the same type of vaccine, because I have tried it, and I do not know how the other vaccines will affect me”* **P18.** *“I prefer the same type of vaccine. According to my experience, I have a son who r two different types of vaccine, and unfortunately, he experienced severe side effects”* **P1.** *“I prefer to continue on the same type because I became fully aware of the potential side effects that I may be exposed to”* **P3.** *“No, I prefer the same type of vaccine that I took because I have tried it and my body may accept it”* **P2.**

The second sub-theme was: ***the lack of awareness about using different types of vaccine.*** Where some of the participants reported that they lack the awareness about the different types of vaccines, thus, they cannot decide which vaccine to receive in the future. *“We have limited knowledge about COVID-19 vaccines, and more information should be provided to the public. What distinguishes these vaccines from one another? I believe we lack sufficient knowledge, but we follow hadiths and gossip, without a medical reference”* **P10.**

The second emerged theme related to the willingness to take the vaccines regularly in the future was: **willingness to receive the vaccine annually.** The participants were asked if they prefer taking the vaccines annually, and it was found that 70.0% of the participants (n = 14) were willing to take the vaccine annually, since they believed that the vaccine is better than the disease itself and help to decrease the aggressive effect of the disease. *“Yes, of course, I will take it, even if it is a different type from the one I previously took, as long as it is allowed by the health organizations, I do not have any problems with that”* **P7.** *“I will take the vaccine annually and I have no objections to taking it on a regular basis”* **P8.** *“Yes, I will take it annually, and I am already planning to take the fourth dose”* **P11.**

## Discussion

In the current qualitative study, five main domains were assessed, evaluating vaccines safety, differences between vaccines, prevention and management of COVID-19 vaccines’ side effects, willingness to report side effects from COVID-19 vaccines, and future willingness to take the vaccine regularly.

The study’s findings showed that most participants believed that COVID-19 vaccines are generally safe to take based on news, social media, and doctors. This encouragement led them to take the vaccines. Governments worldwide have been emphasizing to their populations that COVID-19 vaccines are safe [22]. The side effects of vaccines are less severe than the negative effects of COVID-19, and are considered a natural reaction by the immune system. Other studies have also observed vaccine preference among participants, but for different reasons. For example, participants from Southern Switzerland stated preferred vaccines, because they reduce the need for other protective precautions [23].

This study has shown that the COVID-19 vaccines are not equivalent in terms of safety due to differences in the manufacturer company and the mechanism of action, as each vaccine interacts with the body differently. This finding is consistent with a previously published review article, which indicated that there are several approaches used to develop the COVID-19 vaccines, and that each has its own different advantages and disadvantages [24]. Moreover, a previously published study comparing COVID-19 vaccines found sex differences in efficacy and safety, and that thrombotic side effects were common for the AstraZeneca vaccine [25].

Regarding the prevention and management of COVID-19 vaccines’ side effects, our study found that the majority of the participants took various preventive measures, such as getting tested for COVID-19, avoiding taking the vaccine while having any other infections, taking vitamins, eating healthy food, and engaging in physical activity to boost immunity. Participants believed that these preventive measures would either prevent or at least decrease the side effects associated with the COVID-19 vaccines. This aligns with the awareness campaigns from the Jordanian Ministry of Health and the WHO guidelines [26]. In addition, another study that addressed COVID-19 disease, prevention, and management confirmed this theme and strongly advised and requested all individuals to follow the preventative measures. Otherwise, the situation will be much worse. The authors see this as the best option, since the treatment is not available yet [27]. In China, individual case reports were collected, and the outcomes revealed that preventive measures such as social distancing and quarantine are needed to reduce the spread of the virus [28].

Participants in the present study indicated a willingness to report side effects from COVID-19 vaccines, which varied depending on severity aggressiveness, duration, and novelty of the side effects. It is essential to raise awareness in this situation to encourage people to report any unusual side effects and provide them with easy access to do so. A similar study on vaccines safety recommended paying more attention to and closely monitoring the unusual side effects to determine whether they are related to the vaccines [29]. Worldwide, governments encouraged the public to report any COVID-19 vaccine side effects, and Jordan is no exception. The Jordanian Ministry of Health has asked those who received the COVID-19 vaccine to report any side effects through their platform by clicking on the link included in the message sent to them after receiving the vaccine [30, 31]. Reporting post-vaccination symptoms play a pivotal role in various crucial aspects related to public health. For example, by monitoring vaccine safety, through assessing and analyzing the reported side effects, research and healthcare team can detect unreported or severe side effects that might not have been apparent in the clinical trials. Moreover, transparent reporting of side effects increases public trust in vaccination programs, and decreases public hesitation to receive the vaccine.

This is the first study in Jordan to highlight that most of the participants preferred a vaccine they had previously received. Lacking of knowledge about receiving a different type of vaccine was the major reason for hesitancy among the study participants. Future studies are needed to improve the vaccine acceptance and provide the population with the necessary knowledge about taking different types of vaccines. Furthermore, social media is rife with false information, underscoring the crucial role of the Ministry of Health and health care providers in disseminating accurate evidence and correct information to the public. [22]. In this study the participants’ willingness to receive the vaccine annually was highlighted, as they believed that the vaccine was better than the disease itself and would help decrease its aggressive effects. This finding is consistent with a global survey conducted in 19 countries [32].

The study's main limitation is its low response rate. Many potential participants refused to participate due to concerns about confidentiality, lack of interest, poor knowledge, and objections received from stakeholders in the regulatory authority. All these factors combined limited the scope of the study, and there is a chance that the results reflect the ideal opinion rather than a representative sample of the population. In addition, the use of a non-probability convenience sampling method may affect the representation of different perspectives and experiences related to adverse effects of COVID-19 vaccines.

## Conclusion

The study revealed that most of the participants had trust in the safety of COVID-19 vaccines. The findings highlighted the importance of the Ministry of Health and health care providers’ in raising awareness among the public about the significance of these vaccines, as well as encouraging people to report any unusual side effects. Future studies are needed to address vaccine hesitancy and improve acceptance towards different types of vaccines.

## Data Availability

The authors confirm that the data supporting the findings of this study are available within the article.
